# Aflibercept, ranibizumab, and bevacizumab for macular neovascularization secondary to age-related macular degeneration: a retrospective OCT-angiography study

**DOI:** 10.1186/s40942-025-00740-y

**Published:** 2025-10-16

**Authors:** Marco Lombardo, Carlo Maccauro, Michele D’Ambrosio, Massimo Grossi, Filippo Missiroli, Massimo Cesareo, Federico Ricci

**Affiliations:** Retina Unit, Department of Experimental Medicine, University of Rome Tor Vergata, Viale Oxford 81, 00133 Rome, Italy

**Keywords:** Age-related macular degeneration, AMD, Macular neovascularization, MNV, Anti-VEGF, Aflibercept, Ranibizumab, Bevacizumab, OCT-angiography, OCTA

## Abstract

**Background:**

Intravitreal aflibercept, ranibizumab, and bevacizumab represent the three most widely used anti-VEGF agents for the treatment of neovascular age-related macular degeneration (AMD). The objective of this study is to compare the loading phase effects of these three agents on the macular neovascularization (MNV) area and flow in treatment-naïve eyes affected by neovascular AMD, utilizing optical coherence tomography angiography (OCTA).

**Methods:**

Eighty-four neovascular AMD eyes from eighty-four patients were included in this retrospective study. Twenty-five patients were treated with aflibercept, thirty-four with ranibizumab, and twenty-five with bevacizumab within the initial loading phase preceding a treat-and-extend regimen. All patients underwent a three-monthly injection loading phase and were evaluated at baseline and one month after the loading phase. Best-corrected visual acuity (BCVA), central retinal thickness (CRT), MNV area, and MNV flow area were assessed. MNV parameters were measured using the device-integrated “Flow Area” tool and ImageJ software.

**Results:**

Average baseline BCVA and CRT were 0.60 ± 0.22 logMAR and 343 ± 46 μm, respectively, and did not differ between groups. All three anti-VEGF agents improved anatomical and functional parameters, with no statistically significant differences between treatment groups (*p* > 0.05). Aflibercept showed the most significant CRT reduction (88 μm; *p* < 0.01), while bevacizumab led to the highest BCVA gain (0.15 logMAR; *p* < 0.01). MNV area significantly decreased only in the bevacizumab group (0.26 mm2; *p* < 0.01), and flow area only in the ranibizumab group (0.23 mm2; *p* < 0.05). A strong positive correlation between MNV areas and flow areas was found at baseline (*r* > 0.8) and follow-up (*r* > 0.9).

**Conclusions:**

Aflibercept, ranibizumab, and bevacizumab demonstrated similar efficacy in reducing MNV area and flow and improving visual and anatomical outcomes after a three-injection loading phase. OCTA-derived flow area measurement may be a valuable and easily accessible tool in monitoring early treatment response.

## Background

Age-related macular degeneration (AMD) is a leading cause of irreversible central vision loss among individuals over 60 years old in developed countries. The increasing global prevalence, driven by an aging population, highlights the importance of optimizing treatment strategies and monitoring to address this significant clinical and public health concern. The condition is characterized by a gradual degeneration of the macula, which impairs visual acuity, contrast sensitivity, and reading skills, thereby significantly diminishing the quality of life and independence of patients [1]. AMD has two main forms: the non-neovascular (dry) form, which constitutes about 85% of cases and is characterized by the accumulation of drusen and geographic atrophy in the late stage, and the neovascular (wet or exudative) form, which represents about 15% of cases, but is responsible for the majority of significant vision loss associated with the disease [2].

Neovascular AMD is defined by the development of macular neovascularization (MNV), caused by abnormal blood vessel growth from the choroid through Bruch’s membrane into the subretinal space or below the retinal pigment epithelium. These abnormal vessels tend to leak, bleed, and cause fibrosis, which can ultimately result in photoreceptor degeneration and irreversible central vision loss if not treated [3].

Over the last two decades, intravitreal anti-vascular endothelial growth factor (VEGF) therapy has transformed the management of AMD. Drugs like aflibercept, ranibizumab, and bevacizumab have shown significant effectiveness in slowing disease progression, encouraging the regression of neovascularization, and enhancing or maintaining visual outcomes in large randomized controlled trials [4–6].

These therapies primarily target isoform A of VEGF, a crucial factor in angiogenesis and vascular permeability in AMD, and are generally given as repeated intravitreal injections based on fixed, pro re nata, or treat-and-extend regimens [7]. Aflibercept is a VEGF receptor decoy fusion protein that binds VEGF-A, VEGF-B, and placental growth factor (PlGF); ranibizumab is a humanized monoclonal antibody fragment (Fab) that blocks VEGF-A; bevacizumab is a full-length anti–VEGF-A monoclonal antibody [6].

Optical coherence tomography angiography (OCTA) is a non-invasive imaging technique that allows visualization of retinal and choroidal blood vessels by detecting motion contrast caused by blood flow. Unlike traditional dye-based angiographic methods, such as fluorescein angiography (FA) or indocyanine green angiography (ICGA), OCTA does not require intravenous contrast agents and can capture volumetric data within seconds. This technique relies on repeated B-scans at the same retinal site; the differences in signal intensity between consecutive scans indicate the movement of red blood cells within the vessels. While stationary tissue produces consistent reflectance, areas with blood flow exhibit decorrelation due to the motion of blood cells. OCTA enables the segmentation of the retina vasculature into distinct vascular plexuses, including the superficial capillary plexus, deep capillary plexus, outer retina, and choriocapillaris [8].

Among the most commonly used algorithms in OCTA is the split-spectrum amplitude decorrelation angiography (SSADA), which was designed to enhance flow detection and minimize noise [9].

With the advent of OCTA, it has become possible to facilitate repeated, non-invasive monitoring of MNV in exudative AMD. The effectiveness of each intravitreal injection (IVT) of anti-VEGF drugs can be assessed with accurate follow-up, not only by evaluating intraretinal fluid, subretinal fluid, or pigment epithelium detachment on OCT, but also by analysing segmented en-face OCTA images of the area of interest.

Branching vessels, vascular loops, peripheral anastomotic arcades, and the choriocapillaris dark halo are the most researched OCTA biomarkers for assessing neovascular networks in AMD, particularly in relation to neovascular maturity and activity [10]. OCTA also offered the capability to evaluate the evolution of neovascular membrane area following a single IVT of anti-VEGF [11]. Despite being available for over a decade, OCTA has not been routinely included in most clinical trials assessing the efficacy of intravitreal therapies for AMD. A potential explanation could stem from the lack of automatic and quantifiable OCTA biomarkers for MNV.

The treat-and-extend regimen has been demonstrated to achieve good visual outcomes while minimizing treatment and visit burden. This treatment strategy usually involves an initial loading phase of three IVTs every four weeks, followed by a maintenance phase tailored to the individual patient’s response.

It has been shown that the reduction in MNV area in naïve patients after a monthly loading dose of anti-VEGF does not occur in patients treated with the pro re nata regimen [12]. Indeed, reactive treatment leaves a therapeutic window that could promote the growth of MNV and worsen visual outcomes [13, 14].

The literature indicates that despite being different molecules with distinct molecular targets, the three most used anti-VEGF drugs in clinical practice—bevacizumab, ranibizumab, and aflibercept 2 mg—have similar efficacy in terms of letters gained, notably when administered monthly [15, 16]. Among these, it is essential to note that bevacizumab, along with its already available biosimilars, is currently used off-label in ophthalmology, although it remains widely adopted due to its cost-effectiveness and demonstrated clinical efficacy [17].

Although previous studies have investigated the efficacy of anti-VEGF agents in neovascular AMD, there is limited research directly comparing the effects of different anti-VEGF drugs on the reduction of MNV area as assessed by OCTA. In the present study, we analyse the effectiveness of three different VEGF inhibitors (aflibercept, ranibizumab, and bevacizumab) in reducing the MNV area secondary to AMD measured through OCTA at baseline and after a loading dose (3 monthly injections).

## Methods

The research was conducted in accordance with the Declaration of Helsinki and approved by the Territorial Ethics Committee of Lazio Area 2 (protocol code: 139.25; date of approval: 22/05/2025). This retrospective observational study evaluated the clinical records of patients affected by neovascular AMD at the Tor Vergata Polyclinic Retina Unit in Rome between January 2018 and January 2025. All patient data were fully anonymized before analysis, and no identifying information was retained. All patients provided written informed consent before receiving intravitreal anti-VEGF treatment.

### Inclusion criteria

- Treatment-naïve patients with exudative MNV secondary to AMD.
- Types I and II MNV lesions confirmed by multimodal imaging (FA, ICGA, OCT, and OCTA).
- Complete ophthalmological examination at baseline (within three weeks prior to the first IVT) and after the loading phase (between three and six weeks after the third IVT) of one of the following anti-VEGF drugs: aflibercept 2.00 mg/0.05 mL, (Eylea, Bayer Pharma AG, Berlin, Germany), ranibizumab 0.50 mg/0.05 ml (Lucentis, Novartis Pharma GmbH, Nuernberg, Germany), or bevacizumab 1.25 mg/0.05 ml (Avastin, Roche Holding AG, Basel, Switzerland).
- Best corrected visual acuity (BCVA) assessed with ETDRS charts with baseline BCVA better than 1.0 logMAR.
- FA and ICGA at baseline.
- OCT (Spectralis, Heidelberg Engineering, Heidelberg, Germany) at baseline and after the loading phase.
- OCTA (AngioVue, RTVue XR Avanti OptoVue, Inc., Fremont, CA, USA) at baseline and after the loading phase, with:
  - OCTA macular scans obtained with the high-density (HD) Angio Retina 6 × 6 mm protocol (400 × 400 A-scans).
  - Images with sufficient quality (signal strength ≥ 7/10, absence of significant motion or segmentation artefacts).
  - Lesions had to be fully contained within the 6 × 6 mm scan area.

### Exclusion criteria

- Prior treatment with anti-VEGF agents.
- Type III MNV or polypoidal choroidal vasculopathy.
- Concomitant retinal, vitreoretinal, or optic nerve diseases.
- Advanced lesions that were incapable of functional recovery.
- Incomplete records.

The MNV classification was conducted using multimodal imaging, analysing FA, ICGA, OCT, and OCTA.

To minimize operator influence bias, automated segmentation of the “choriocapillaris” and the “outer retina” was employed for type I and II lesions at baseline and after the loading phase. Only en-face images where the neovascular network was visible and consistent with FA and ICGA examinations were included.

The “ImageJ—Image Processing & Analysis in Java” software was used to calculate the area, allowing conversion of image dimensions from pixels to millimetres and the calculation of the surface of the selected area.

En-face OCTA slabs were exported as 8-bit TIFF files at native resolution with scale metadata preserved. Firstly, 600 pixels were set in height (height pixels) and width (width pixels) to obtain an enlarged image. The conversion to millimetres was achieved by setting the image edge to 6 mm using the “Analyze – Set Scale” function; subsequently, the “Polygon Selection” function was used to outline the area of interest, following the entire boundary of the MNV. Finally, through the “Analyze – Measure” function, the software calculated the area of the selected portion.

To calculate the baseline and follow-up flow area, the “Flow Area” function of the OCTA software was utilized after delineating the neovascular network through the “Contour-Draw” function, which followed the shape of the MNV similarly to the “ImageJ” software. The metric is semi-automated once the perimeter is defined. This is conceptually distinct from the ImageJ area (binary lesion area) and captures the perfused area within the user-defined boundary.

Two experienced ophthalmologists (F.M. and M.C.) independently performed these measurements in a masked modality for all randomly presented baseline and follow-up en-face images. No repeated measurements were performed. In cases of disagreement, a senior retina specialist (F.R.) reviewed the images and provided the final adjudication.

### Statistical analysis

The following data were collected at baseline and post-loading phases: BCVA expressed in logMAR units, central retinal thickness (CRT) expressed in microns, MNV assessed using ImageJ software, and MNV flow area. Clinical Data were entered into an Excel spreadsheet (Microsoft, Redmond, Washington, USA). Statistical analysis was then conducted using the Statistical Package for the Social Sciences (SPSS, Chicago, Illinois, USA). No a priori sample size calculation was performed due to the retrospective and exploratory nature of the study. The sample was determined by the number of eligible patients meeting the inclusion criteria within the defined study period.

The Kolmogorov-Smirnov test was used to assess the distribution of the variables. Normally distributed variables were presented as mean ± standard deviation and range (min., max.). Frequency data were presented as percentages. A one-way ANOVA test was conducted for between-group comparisons of normally distributed variables (age, BCVA, CRT, MNV area, and MNV flow area). Categorical occurrence data (gender and MNV type) were compared between groups using the chi-square test. Baseline and post-loading changes for each parameter were analysed within each treatment group using paired t-tests.

Category outcome classes were defined for each clinical parameter to assess the distribution of therapeutic outcomes among the three treatment groups (aflibercept, ranibizumab, and bevacizumab). BCVA changes were classified as improved (gain ≥ 0.3 logMAR), stable (change within ± 0.2 logMAR), or worsened (loss ≥ 0.3 logMAR). CRT changes were categorized as decreased (reduction ≥ 50 μm), stable (change within ± 50 μm), or increased (increase ≥ 50 μm). Changes in MNV area and flow area were considered reduced (≥ 5% reduction), stable (change within ± 5%), or increased (≥ 5% increase). The proportions of patients in each outcome category were calculated for each treatment group, and differences between groups were assessed using the chi-square test of independence. The effectiveness of each drug in reducing BCVA, CRT, MNV area, and MNV flow area was also compared between groups by analysing the absolute and percentage changes from baseline using one-way ANOVA. To assess the relationship between MNV area and MNV flow area, the Pearson correlation coefficient was calculated separately for each treatment group at baseline and after the loading phase. A p-value of less than 0.05 was considered statistically significant.

## Results

Eighty-four neovascular AMD eyes from eighty-four patients were included in the study. Twenty-five patients were treated with aflibercept, thirty-four with ranibizumab, and twenty-five with bevacizumab. The age, gender, and MNV type distributions were similar between groups (*p* > 0.05). Baseline characteristics (BCVA, CRT, MNV area, and MNV flow area) did not differ significantly among the three groups examined (*p* > 0.05) (Table 1).

**Table 1:** Baseline characteristics among the three treatment groups

Parameter	Aflibercept	Ranibizumab	Bevacizumab	*p*-value
Age	77.4 ± 4.0	79.1 ± 4.1	77.6 ± 5.4	0.281
Female gender (%)	52.0%	44.1%	52.0%	0.778
MNV type 1 (%)	36.0%	47.1%	56.0%	0.364
BCVA (logMAR)	0.63 ± 0.24	0.62 ± 0.20	0.56 ± 0.20	0.464
CRT (micron)	352 ± 52	342 ± 43	334 ± 39	0.351
MNV area (mm²)	1.95 ± 1.00	1.66 ± 1.34	1.79 ± 1.10	0.656
MNV flow area (mm²)	1.56 ± 0.90	1.29 ± 1.06	1.33 ± 0.89	0.543

MNV: macular neovascularization; BCVA: best corrected visual acuity; CRT: central retinal thickness

Table 2 summarizes the intra-group analysis, comparing baseline measurements to post-loading values within each treatment group.

**Table 2:** Intra-group analysis comparing baseline parameters to those measured after the loading phase for each drug

Parameter	Drug	Baseline (mean ± SD)	Post-loading (mean ± SD)	*p*-value
BCVA (logMAR)	Aflibercept	0.63 ± 0.24	0.60 ± 0.32	0.543
	Ranibizumab	0.62 ± 0.2	0.54 ± 0.26	< 0.05
	Bevacizumab	0.56 ± 0.2	0.41 ± 0.24	< 0.01
CRT (µm)	Aflibercept	351.96 ± 52.15	263.96 ± 67.98	< 0.01
	Ranibizumab	342.32 ± 42.69	278.09 ± 84.65	< 0.01
	Bevacizumab	333.56 ± 38.85	269.96 ± 83.87	< 0.01
MNV area (mm²)	Aflibercept	1.95 ± 1.0	1.81 ± 1.09	0.059
	Ranibizumab	1.66 ± 1.34	1.46 ± 1.14	0.064
	Bevacizumab	1.79 ± 1.1	1.53 ± 1.02	< 0.01
MNV flow area (mm²)	Aflibercept	1.56 ± 0.9	1.29 ± 0.9	0.055
	Ranibizumab	1.29 ± 1.06	1.06 ± 1.02	< 0.05
	Bevacizumab	1.33 ± 0.89	1.14 ± 0.92	0.148

BCVA: best corrected visual acuity; CRT: central retinal thickness; MNV: macular neovascularization; SD: standard deviation

Table 3 presents a summary of the inter-group analysis, in which the post-loading effects of aflibercept, ranibizumab, and bevacizumab were compared in terms of absolute values, difference between baseline and post-loading (Δ), and percentage (%) of reduction.

**Table 3:** Inter-group analysis of the post-loading effects among the three drugs

Parameter	Drug	Post-loading (mean ± SD)	*p*-value (post-loading)	Δ (mean ± SD)	*p*-value (Δ)	% reduction (mean ± SD)	*p*-value (% reduction)
BCVA (logMAR)	Aflibercept	0.60 ± 0.32	0.057	0.03 ± 0.26	0.198	0.50 ± 53.88	0.220
	Ranibizumab	0.54 ± 0.26		0.08 ± 0.20		13.10 ± 35.77	
	Bevacizumab	0.41 ± 0.24		0.15 ± 0.22		23.21 ± 49.27	
CRT (µm)	Aflibercept	263.96 ± 67.98	0.794	88.00 ± 49.29	0.260	25.48 ± 15.04	0.420
	Ranibizumab	278.09 ± 84.65		64.24 ± 56.62		19.84 ± 16.97	
	Bevacizumab	269.96 ± 83.87		63.60 ± 75.26		19.32 ± 23.41	
MNV area (mm²)	Aflibercept	1.81 ± 1.09	0.472	0.14 ± 0.35	0.697	9.40 ± 18.25	0.727
	Ranibizumab	1.46 ± 1.14		0.20 ± 0.60		9.57 ± 22.32	
	Bevacizumab	1.53 ± 1.02		0.26 ± 0.43		13.35 ± 18.88	
MNV flow area (mm²)	Aflibercept	1.29 ± 0.90	0.665	0.27 ± 0.67	0.883	11.19 ± 40.57	0.764
	Ranibizumab	1.06 ± 1.02		0.23 ± 0.56		8.41 ± 160.80	
	Bevacizumab	1.14 ± 0.92		0.18 ± 0.62		8.51 ± 73.58	

BCVA: best corrected visual acuity; CRT: central retinal thickness; MNV: macular neovascularization; SD: standard deviation

**Fig. 1:**
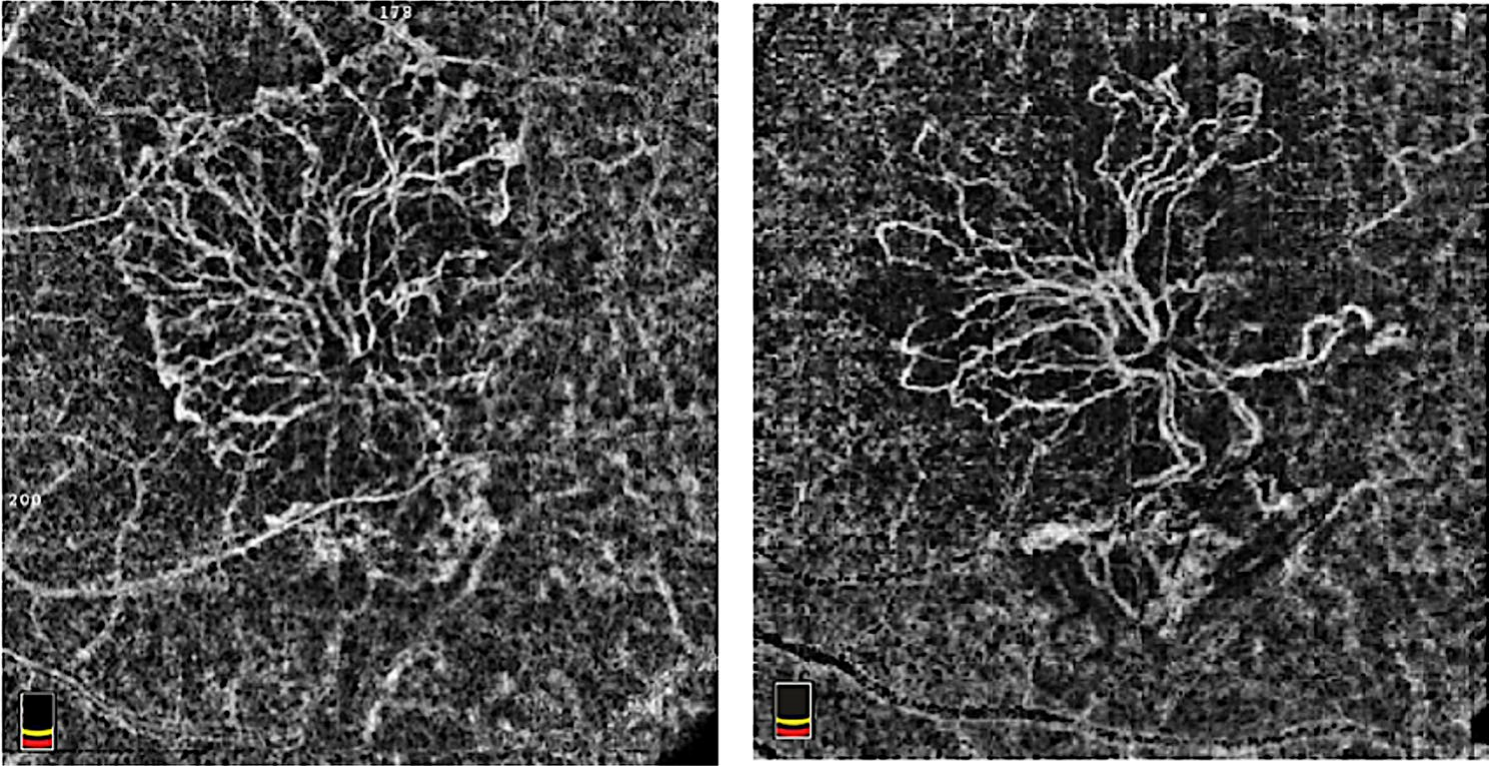
Optical coherence tomography angiography (OCTA) of the macular neovascularization (MNV). The figure shows a representative OCTA image of the MNV at baseline (left) and the regression of the neovascular network after the loading phase of the anti-VEGF intravitreal injections (right)

All three anti-VEGF treatments resulted in improvements in BCVA, CRT, MNV area, and MNV flow area. Figure 1 illustrates representative clinical examples of MNV evolution following intravitreal anti-VEGF treatment.

Regarding BCVA, most patients exhibited functional stability, with percentages ranging from 72.0% to 79.4% across the three treatment groups. Visual improvement was observed in 24.0% of patients treated with aflibercept, 28.0% of those receiving bevacizumab, and 20.6% of patients treated with ranibizumab. Visual deterioration was reported solely within the aflibercept group (4.0%), while no patients in either the ranibizumab or bevacizumab groups experienced a loss in BCVA ≥ 0.3 logMAR. In terms of CRT, a reduction of ≥ 50 μm was more prevalent in the aflibercept group (92.0%), compared to 68.0% of patients treated with bevacizumab and 67.6% with ranibizumab. A stable CRT was documented in 29.4% of patients receiving ranibizumab, 20.0% of those treated with bevacizumab, and 4.0% of patients in the aflibercept group. While increases in CRT were infrequent, they were more pronounced in the bevacizumab group (aflibercept: 4%; ranibizumab: 2.9%; bevacizumab: 12.0%). Concerning the MNV area, nearly half of the patients demonstrated a stable lesion (change within ± 5%), respectively 48.0% for aflibercept, 56.0% for bevacizumab, and 55.9% for ranibizumab. A ≥ 5% decrease was noted in 40.0% of patients treated with aflibercept and bevacizumab and 29.4% of patients treated with ranibizumab. The MNV area was increased in 12.0% of patients receiving aflibercept, 14.7% of those treated with ranibizumab, and 4.0% of those treated with bevacizumab. The OCTA MNV flow area showed a trend similar to the ImageJ area: the proportion of patients with a stable flow was 52.0% for the aflibercept group, 60.0% for bevacizumab, and 61.8% for ranibizumab. A lower percentage of patients experienced a reduction of 5% or more, ranging from 26.5% to 36.0%, and a limited proportion of patients showed an increase in the flow area, from 8.0% to 12.0%.

However, the intra-group analysis revealed some variability in the statistical significance of these changes: only ranibizumab and bevacizumab showed a significant improvement in BCVA, while the reduction in the MNV area was statistically significant only in the bevacizumab group, and the MNV flow area was significant only in the ranibizumab group (Table 2).

The inter-group analysis, conducted on absolute values and reduction percentages, did not show statistically significant differences between the three drugs for any of the considered parameters (Table 3).

These results indicate that, although the extent of response may vary within individual groups, the three drugs demonstrate comparable clinical efficacy in reducing neovascular activity, both from a functional and structural perspective.

**Fig. 2:**
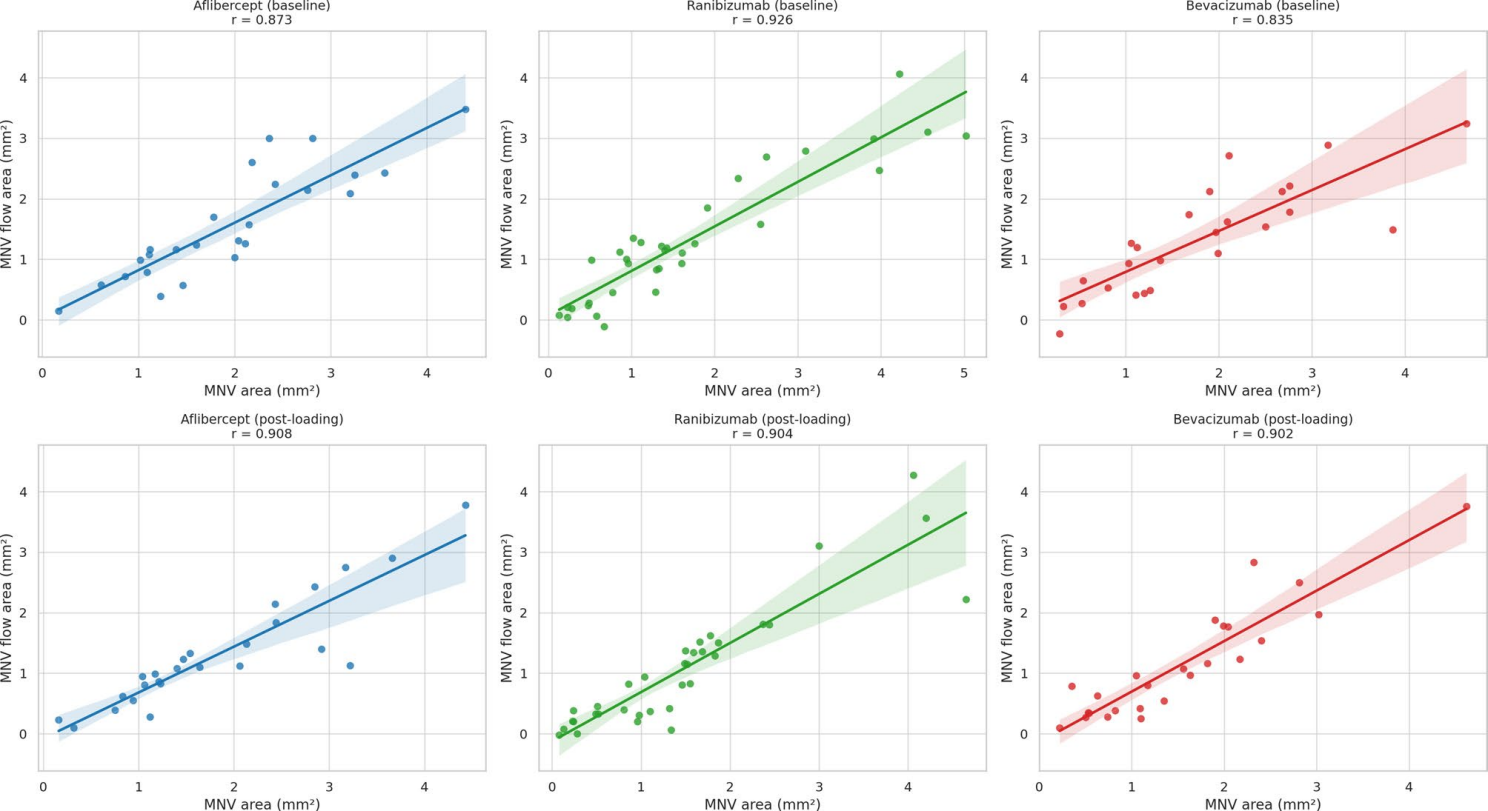
Correlations between the macular neovascularization (MNV) area and the MNV flow area. The scatter plot illustrates the strong correlations between the MNV area measured with ImageJ software and the MNV flow area directly measured with the OCTA device

Finally, the MNV area values calculated using ImageJ software showed a strong direct correlation with the MNV flow area calculated through the included OCTA tool (Fig. 2).

The proportion of patients with no fluid (absence of macular intraretinal and subretinal fluid) following the loading phase was 40%, 41%, and 48% for aflibercept, ranibizumab, and bevacizumab, respectively. In the remaining patients, the amount of fluid decreased, reflecting the CRT, with no cases of worsening or new-onset fluid observed after the loading phase. Specifically, the percentage of patients with intraretinal fluid decreased from 72% (baseline) to 16% (after loading phase) in the aflibercept-treated group, from 59% to 12% in the ranibizumab-treated group, and from 52% to 16% in the bevacizumab-treated group. On the other hand, the percentage of patients with sub-retinal fluid decreased from 76% to 20% in the aflibercept-treated group, from 59% to 23% in the ranibizumab-treated group, and from 60% to 20% in the bevacizumab-treated group.

## Discussion

This study presents a comparative evaluation of the loading phase efficacy of aflibercept, ranibizumab, and bevacizumab under a treat-and-extend regimen in reducing the MNV area and flow in treatment-naïve patients with neovascular AMD using OCTA.

Our findings confirm that all three anti-VEGF agents effectively promote anatomical and functional improvements after the initial loading phase, with no statistically significant differences observed between the intravitreal drugs in inter-group comparisons (*p* > 0.05, Table 3).

Despite their different molecular structures and mechanisms of VEGF inhibition, aflibercept, ranibizumab, and bevacizumab produced similar improvements in key parameters, including BCVA, CRT, MNV area, and flow area. Minor differences between groups, such as the higher CRT reduction with aflibercept and greater BCVA improvement with bevacizumab, did not reach statistical significance in inter-group analysis (*p* > 0.05, Table 3).

Nonetheless, it is important to recognize the different pharmacodynamic and pharmacokinetic profiles of these molecules [18].

Aflibercept achieved the most significant reduction in CRT, although this did not result in significant visual improvement. This discrepancy could be due to various confounding factors, such as individual variations in disease duration or existing photoreceptor damage, which were not part of the initial evaluation planned in this study design. Moreover, it is well-known that CRT reduction does not consistently correlate with functional enhancement, particularly in patients with limited visual function [19].

Bevacizumab, despite its off-label use in ophthalmology, showed the most substantial improvement in visual acuity and a significant reduction in MNV area.

On the other hand, ranibizumab resulted in a significant decrease in MNV flow area but not in total lesion size, suggesting that flow-based assessments might serve as a more sensitive early indicator of vascular response compared to area measures alone.

The results of intra-group analysis reveal significant improvements in BCVA for both the ranibizumab and bevacizumab groups, but not for aflibercept, which may appear counterintuitive, given aflibercept’s superior performance in CRT reduction. A possible explanation could lie in the reduced functional reserve of the patients treated with aflibercept or baseline differences not captured in the statistical model, such as fluid distribution, presence of fibrosis, chronicity, or patient comorbidities.

However, all treatments displayed favourable trends, suggesting that a longer follow-up might be necessary to detect broader statistical differences.

A strong correlation was observed between MNV area and MNV flow area across all treatment groups at both baseline and after the loading phase. Our findings support the practicality of using the OCTA device’s built-in “Flow Area” tool as a rapid and non-invasive method for monitoring treatment response in clinical settings, potentially reducing reliance on labour-intensive post-processing methods, such as ImageJ.

Approximately half of the patients in each group demonstrated stable MNV areas and flow values after the loading phase. This suggests that, while the initial anti-VEGF response is generally favourable, the degree of neovascular remodelling may be limited in a significant proportion of patients over the short term. On the other hand, other qualitative features in OCTA, such as branching vessels or vascular loops, may have changed without modifying the overall MNV area. Even a stable inactive lesion typically still shows a detectable area on OCTA.

In line with previous studies, our results support that early anti-VEGF treatment results in significant changes in neovascular structure, but that complete regression or inactivity is rarely achieved within the first three months [20]. This concept is further supported by the significant percentage of patients with residual fluid after the loading phase found in our study. This underlines the need for continuous treatment and strengthens the case for proactive regimens, such as “treat-and-extend”.

Prior studies have reported that aflibercept may result in greater resolution of subretinal fluid and improved outer retinal integrity restoration compared to ranibizumab or bevacizumab, especially in treatment-naïve eyes [21]. However, comparative analyses of retinal layer thicknesses using OCT have shown that all three drugs produce similar changes in the inner retinal layers, such as the ganglion cell layer and retinal nerve fibre layer, with no significant differences between agents [22, 23]. Our study did not stratify outcomes by retinal layer; however, it would be interesting to explore in future research whether OCTA could also aid in assessing the vascularization of the inner retinal layers in response to anti-VEGF therapy.

Furthermore, evidence suggests that qualitative changes in MNV morphology, such as vessel rarefaction, peripheral loop pruning, and reduced capillary complexity, may occur before or even without area reduction [24].

In the broader literature context, our results align with previous comparative studies, which found no significant differences in visual outcomes among the three examined drugs [25–27]. However, our focus on anatomical changes using OCTA provides an updated perspective, reflecting the growing clinical emphasis on microvascular analysis in managing AMD. Furthermore, this study offers novel insights into the early morphological changes in MNV in response to anti-VEGF therapy, an area that remains under active investigation.

The potential clinical implications of this study may be significant. The similar short-term efficacy profiles of the agents studied may allow clinicians a greater flexibility in the treatment choice, prioritizing factors such as cost and availability when selecting the most appropriate therapy. The distinct intra-group variabilities observed in the study may suggest a need to identify specific biomarkers or lesion characteristics that can predict which patients are most likely to respond optimally to a particular anti-VEGF agent, thereby allowing for more tailored and effective treatment strategies.

Some limitations of this research should be acknowledged. The study did not fully assess the entire treat-and-extend regimen, as it only examined the three-injection loading phase. Treatment-naïve patients often respond well initially, but differences in durability and the need for retreatment may emerge over time, and long-term outcomes may diverge between drugs as the disease progresses. The retrospective nature of the study introduced potential selection biases. While the randomization of image analysis and the blinding of graders reduced observer bias, OCTA devices featuring automatic MNV area quantification may provide a more objective standardization. The relatively small sample size may have limited the power to detect subtle inter-drug differences. Lastly, the short follow-up duration limited conclusions regarding the long-term sustainability of the observed anatomical and functional improvements.

Future prospective studies with larger cohorts and standardized imaging protocols are necessary to validate these findings. Similar works could be implemented in the near future using more recently approved intravitreal drugs such as brolucizumab, faricimab, and aflibercept 8 mg. We hope that the integration of supplementary imaging biomarkers, including vessel density, leakage quantification, and choriocapillaris perfusion, potentially facilitated by novel advanced artificial intelligence software [28], may provide deeper insights into the dynamics of vascular remodelling during anti-VEGF therapy.

## Conclusions

This study highlighted that aflibercept, ranibizumab, and bevacizumab provided similar short-term anatomical and functional improvements in treatment-naïve neovascular AMD during the intravitreal loading phase strategy. Our findings may endorse increased flexibility in clinical decision-making; given that all three agents offered comparable advantages following the loading phase, considerations such as drug availability, cost, and patient-specific factors may be prioritized in the therapy selection. OCTA-based assessments have been shown to be effective for monitoring macular neovascularization. These results underscore the clinical importance of OCTA in treatment monitoring, but future prospective studies are essential to support these findings. The use of OCTA-derived metrics in future clinical trials evaluating anti-VEGF therapy could be beneficial. Establishing standardized definitions of lesion activity based on blood flow and morphology, rather than solely on the presence of fluid, has the potential to enhance the personalization of treatment and facilitate the early identification of non-responders during the progression of the disease. In summary, our findings reinforce the role of OCTA as a valuable adjunct in the management of neovascular AMD; they also provide a basis for future prospective studies aimed at optimizing treatment selection and monitoring strategies in clinical practice.

## Data Availability

The datasets used and analysed during the current study are available from the corresponding author on reasonable request.

## Abbreviations

OCTA: Optical coherence tomography angiography
OCT: Optical coherence tomography
MNV: Macular neovascularization
AMD: Age-related macular degeneration
IVT: Intravitreal injection
VEGF: Vascular endothelial growth factor
PlGF: Placental Growth Factor
BCVA: Best corrected visual acuity
FA: Fluorescein angiography
ICGA: Indocyanine green angiography
CRT: Central retinal thickness

## References

[1] Wong WL, Su X, Li X, Cheung CMG, Klein R, Cheng CY, et al. Global prevalence of age-related macular degeneration and disease burden projection for 2020 and 2040: a systematic review and meta-analysis. Lancet Glob Health. 2014;2(2):e106–16. 25104651 10.1016/S2214-109X(13)70145-1

[2] Ferris FL, Wilkinson CP, Bird A, Chakravarthy U, Chew E, Csaky K, et al. Clinical classification of Age-related macular degeneration. Ophthalmology. 2013;120(4):844–51. 23332590 10.1016/j.ophtha.2012.10.036 PMC11551519

[3] Spaide RF, Jaffe GJ, Sarraf D, Freund KB, Sadda SR, Staurenghi G, et al. Consensus nomenclature for reporting neovascular Age-Related macular degeneration data. Ophthalmology. 2020;127(5):616–36. 31864668 10.1016/j.ophtha.2019.11.004 PMC11559632

[4] Rosenfeld PJ, Brown DM, Heier JS, Boyer DS, Kaiser PK, Chung CY, et al. Ranibizumab for neovascular Age-Related macular degeneration. N Engl J Med. 2006;355(14):1419–31. 17021318 10.1056/NEJMoa054481

[5] Ranibizumab and Bevacizumab for Neovascular Age-Related Macular Degeneration. N Engl J Med. 2011;364(20):1897–908. 21526923 10.1056/NEJMoa1102673 PMC3157322

[6] Heier JS, Brown DM, Chong V, Korobelnik JF, Kaiser PK, Nguyen QD, et al. Intravitreal Aflibercept (VEGF Trap-Eye) in wet Age-related macular degeneration. Ophthalmology. 2012;119(12):2537–48. 23084240 10.1016/j.ophtha.2012.09.006

[7] Jaffe GJ, Ciulla TA, Ciardella AP, Devin F, Dugel PU, Eandi CM, et al. Dual antagonism of PDGF and VEGF in neovascular Age-Related macular degeneration. Ophthalmology. 2017;124(2):224–34. 28029445 10.1016/j.ophtha.2016.10.010

[8] De Carlo TE, Romano A, Waheed NK, Duker JS. A review of optical coherence tomography angiography (OCTA). Int J Retina Vitr. 2015;1(1):5. 10.1186/s40942-015-0005-8 PMC5066513 27847598

[9] Jia Y, Tan O, Tokayer J, Potsaid B, Wang Y, Liu JJ, et al. Split-spectrum amplitude-decorrelation angiography with optical coherence tomography. Opt Express. 2012;20(4):4710. 22418228 10.1364/OE.20.004710 PMC3381646

[10] Rispoli M, Cennamo G, Antonio LD, Lupidi M, Parravano M, Pellegrini M, et al. Practical guidance for imaging biomarkers in exudative age-related macular degeneration. Surv Ophthalmol. 2023;68(4):615–27. 36854371 10.1016/j.survophthal.2023.02.004

[11] Muakkassa NW, Chin AT, De Carlo T, Klein KA, Baumal CR, Witkin AJ, et al. Characterizing the effect of anti-vascular endothelial growth factor therapy on treatment-naive choroidal neovascularization using optical coherence tomography angiography. Retina. 2015;35(11):2252–9. 26457400 10.1097/IAE.0000000000000836

[12] Matsumoto H, Hoshino J, Nakamura K, Akiyama H. Two-year outcomes of treat-and-extend regimen with intravitreal Brolucizumab for treatment-naïve neovascular age-related macular degeneration with type 1 macular neovascularization. Sci Rep. 2023;13(1):3249. 36828853 10.1038/s41598-023-30146-5 PMC9958126

[13] Miere A, Oubraham H, Amoroso F, Butori P, Astroz P, Semoun O, et al. Optical coherence tomography angiography to distinguish changes of choroidal neovascularization after Anti-VEGF therapy: monthly loading dose versus pro re Nata regimen. J Ophthalmol. 2018;2018:1–7. 10.1155/2018/3751702 PMC5817334 29507810

[14] Cozzi M, Monteduro D, Esposito RA, Spooner KL, Fraser-Bell S, Staurenghi G, et al. Lesion area progression in eyes with neovascular age-related macular degeneration treated using a proactive or a reactive regimen. Eye. 2024;38(1):161–7. 37393395 10.1038/s41433-023-02652-3 PMC10764886

[15] Berg K, Pedersen TR, Sandvik L, Bragadóttir R. Comparison of Ranibizumab and bevacizumab for neovascular Age-Related macular degeneration according to LUCAS Treat-and-Extend protocol. Ophthalmology. 2015;122(1):146–52. 25227499 10.1016/j.ophtha.2014.07.041

[16] Schmid MK, Bachmann LM, Fäs L, Kessels AG, Job OM, Thiel MA. Efficacy and adverse events of aflibercept, Ranibizumab and bevacizumab in age-related macular degeneration: a trade-off analysis. Br J Ophthalmol. 2015;99(2):141–6. 25271911 10.1136/bjophthalmol-2014-305149

[17] Solomon SD, Lindsley K, Vedula SS, Krzystolik MG, Hawkins BS. Anti-vascular endothelial growth factor for neovascular age-related macular degeneration. Cochrane Eyes and Vision Group, editor. Cochrane Database Syst Rev. 2019 Mar 4;2019(3) Available from: 10.1002/14651858.CD005139.pub4 PMC6419319 30834517

[18] Stewart MW. Pharmacokinetics, pharmacodynamics and pre-clinical characteristics of ophthalmic drugs that bind VEGF. Expert Rev Clin Pharmacol. 2014;7(2):167–80. 24483136 10.1586/17512433.2014.884458

[19] Maguire MG, Martin DF, Ying G, shuang, Jaffe GJ, Daniel E, Grunwald JE, et al. Five-Year outcomes with Anti–Vascular endothelial growth factor treatment of neovascular Age-Related macular degeneration. Ophthalmology. 2016;123(8):1751–61. 27156698 10.1016/j.ophtha.2016.03.045 PMC4958614

[20] Mettu PS, Allingham MJ, Cousins SW. Incomplete response to Anti-VEGF therapy in neovascular AMD: exploring disease mechanisms and therapeutic opportunities. Prog Retin Eye Res. 2021;82:100906. 33022379 10.1016/j.preteyeres.2020.100906 PMC10368393

[21] Coscas F, Coscas G, Lupidi M, Dirani A, Srour M, Semoun O, et al. Restoration of outer retinal layers after Aflibercept therapy in exudative AMD: prognostic value. Investig Opthalmology Vis Sci. 2015;56(6):4129. 10.1167/iovs.15-16735 26114491

[22] Demir N, Sevincli S, Kayhan B, Sonmez M. Anatomical effects of intravitreal anti-vascular endothelial growth factor injections on inner layers of the lesion-free retina. Cutan Ocul Toxicol. 2021;40(2):135–9. 33944638 10.1080/15569527.2021.1919136

[23] Abu Dail Y, Seitz B, Sideroudi H, Abdin AD. Impact of intravitreal ranibizumab, Aflibercept and bevacizumab on retinal ganglion cell and nerve fibre layer thickness in neovascular age-related macular degeneration. Acta Ophthalmol (Copenh). 2023;101(3):330–41. 10.1111/aos.15283 36345883

[24] Pilotto E, Frizziero L, Daniele AR, Convento E, Longhin E, Guidolin F, et al. Early OCT angiography changes of type 1 CNV in exudative AMD treated with anti-VEGF. Br J Ophthalmol. 2019;103(1):67–71. 29567794 10.1136/bjophthalmol-2017-311752

[25] Freund KB, Korobelnik JF, Devenyi R, Framme C, Galic J, Herbert E, et al. TREAT-AND-EXTEND REGIMENS WITH ANTI-VEGF AGENTS IN RETINAL DISEASES: A literature review and consensus recommendations. Retina. 2015;35(8):1489–506. 26076215 10.1097/IAE.0000000000000627

[26] Matonti F, Korobelnik JF, Dot C, Gualino V, Soler V, Mrejen S, et al. Comparative effectiveness of intravitreal Anti-Vascular endothelial growth factor therapies for managing neovascular Age-Related macular degeneration: A Meta-Analysis. J Clin Med. 2022;11(7):1834. 35407439 10.3390/jcm11071834 PMC8999505

[27] Parravano M, Viola F, Nicolò M, Vujosevic S, Bianchino L, Sicari E, et al. Real-world evidence of anti-VEGF therapies in patients with neovascular age-related macular degeneration in Italy: the RADIANCE study. Eur J Ophthalmol. 2025;11206721241310628. 10.1177/11206721241310628 PMC12166140 39901532

[28] Schranz M, Bogunovic H, Deak G, Sadeghipour A, Reiter GS, Schmidt-Erfurth U. Linking disease activity with optical coherence tomography angiography in neovascular age related macular degeneration using artificial intelligence. Sci Rep. 2024;14(1):19278. 39164449 10.1038/s41598-024-70234-8 PMC11336074

